# Broad phenotypic spectrum in familial adenomatous polyposis; from early onset and severe phenotypes to late onset of attenuated polyposis with the first manifestation at age 72

**DOI:** 10.1186/1471-2350-9-101

**Published:** 2008-11-26

**Authors:** Mef Nilbert, Ulf Kristoffersson, Mats Ericsson, Oskar Johannsson, Eva Rambech, Peter Mangell

**Affiliations:** 1 Department of Oncology, Institute of Clinical Sciences, Lund University, Sweden; 2 Clinical Research Centre, Hvidovre Hospital, Copenhagen University, Denmark; 3 Department of Clinical Genetics, Lund University Hospital, Sweden; 4 Department of Surgery, Kristianstad Hospital, Kristianstad, Sweden; 5 Department of Medical Oncology, University Hospital of Iceland, Reykjavik, Iceland; 6 Department of Surgery, Malmö University Hospital, Sweden

## Abstract

**Background:**

Familial adenomatous polyposis (FAP) is typically characterized by multiple colonic polyps and frequent extracolonic features. Whereas the number of colonic polyps has been linked to the *APC *gene mutation, possible genotype-phenotype correlations largely remain to be defined for the extracolonic manifestations.

**Methods:**

Full genomic sequencing combined with multiplex ligation-dependent probe amplification was used to identify *APC *gene mutations, which were correlated to the clinical presentations.

**Results:**

10 novel *APC *gene mutations were identified in 11 families. A broad spectrum of extracolonic manifestations was identified in most of these individuals. Two sisters with an insertion in codon 528 (c.1582_1583insGC) both showed severe phenotypes with classical polyposis, upper gastrointestinal polyps and thyroid cancer. A woman with a 3'*APC *mutation (c.5030_5031insAA) developed colon cancer at age 72 as the first manifestation of attenuated FAP.

**Conclusion:**

With an increasing number of FAP families diagnosed, a broad and variable tumor spectrum and a high frequency of extracolonic manifestations are gradually recognized. We report novel *APC *mutations and present two FAP cases that suggest familial aggregation of thyroid cancer and demonstrate the need to consider attenuated FAP also among elderly patients with colon cancer.

## Background

Familial adenomatous polyposis (FAP) affects about 1/13–18000 individuals, is characterized by development of multiple colonic polyps and causes less than 1% of colorectal cancer [1,2]. *APC *encodes for a 2843 amino acid protein that contains a variety of functional domains involved in transcription, cell cycle control, migration, differentiation, and apoptosis. Mutations in *APC *were demonstrated in 1991 and to date some 800 mutations have been identified [3,4]. Frameshift mutations predominate and nonsense mutations are found in one third of the cases, whereas large deletions and missense mutations represent rare causes of FAP [5]. A mutation cluster region in the 5'end of exon 15 of the *APC *gene has been identified and the two most frequent mutations, which account for 11–17% of the germline alterations, affect codons 1061 and 1309 [5].

The severity of colonic polyposis has been linked to the genotype. Mutations between *APC *codons 1250 and 1464 cause profuse polyposis, generally with > 5000 polyps, and the recurrent codon 1309 mutation is associated with early onset and development of thousands of polyps [6,7]. The classical or intermediate phenotype is associated with mutations between codons 157 (in exon 4) and 1595 (in exon 15), whereas mutations before codon 157, in the alternatively spliced region of exon 9, and after codon 1595 cause attenuated FAP (AFAP) [8,9].

Extracolonic manifestations are estimated to develop in 70% of FAP patients [6]. Except for an association between mutations in codons 311–1444 and congenital hypertrophy of the retinal pigment epithelium (CHRPE), extracolonic manifestations have been described in conjunction with mutations throughout the *APC *gene [6,7,10]. We present new *APC *mutations and specifically discuss two cases that demonstrate the broad phenotypic spectrum in FAP; two sisters who concordantly developed classical polyposis, upper gastrointestinal polyps, and thyroid cancer and a woman with a 3'*APC *mutation who presented with the first symptom of FAP at age 72.

## Methods

All patients underwent genetic counseling and *APC *mutation testing because of clinical suspicion of FAP. The study complied with Ethics guidelines and the samples were obtained as part of a clinical routine analysis with informed consent. Totally, 15 individuals with clinical FAP manifestations were subjected to mutation analysis, which applied DNA sequencing of exons 1–14 and the protein truncation test (PTT) to screen for truncating mutations in exon 15 [for details see [11]]. Analysis for large deletions was performed using the multiplex ligation-dependent probe amplification (MLPA) assay in cases where sequencing did not identify any mutation [for details see [12]].

## Results

Totally, 15 individuals with clinical FAP were subjected to mutation analysis and *APC *gene mutations were identified in 11 individuals/families, whereas no mutation could be identified in 4 individuals. Clinical characteristics of the cases in which *APC *gene mutations were identified are summarized in table 1. The mutations included 4 small insertions/deletions, 3 nonsense mutations, 2 splice site mutations, and 2 whole-gene deletions (detected by MLPA in cases A37 and A40). 5 individuals reported no family history of polyposis/colorectal cancer. Among patients with FAP-predisposing *APC *gene mutations, colonic adenomas were diagnosed at surveillance or because of symptoms at mean 32 (14–72) years of age. Two individuals were diagnosed with colonic polyposis and synchronous colon cancer at ages 45 and 72, respectively. Duodenal polyps were diagnosed in 9 individuals at mean 40 (24–73) years of age and gastric polyps developed in 7 individuals at mean 40 (26–73) years of age. Desmoid tumors of the abdominal wall and the retroperitoneum developed in two patients at ages 20 and 73 and were associated with mutations in exon 15.

Phenotypic similarity was demonstrated in two sisters (A46-1 and A46-2) with an insertion (c.1582_1583insGC) in an area of the *APC *gene associated with intermediate polyposis. They had a family history of FAP with a mother diagnosed with colonic and duodenal polyposis who later developed a duodenal carcinoma from which she died. Both sisters developed colon polyposis with the first adenomas diagnosed at ages 13 and 18. At ages 18 and 28, respectively, they were diagnosed with papillary thyroid cancer and underwent radical surgery. Upper gastrointestinal manifestations later developed with duodenal polyps diagnosed at ages 27 and 28 and gastric polyps at ages 27 and 40.

**Table 1:** Summary of genotypes and phenotypes

Family number	*APC *gene mutation	Consequence	Type colonic polyposis (A, P, C)	Colonic adenoma age	Duodenal adenoma age	Gastric adenoma age	Other tumor type and location, Age	Family origin
A43/M	c.824delA	p.N276fs	C	32	-	-	adenoma, ureter/32	Sweden
A8/F	c.1120G>T	p.S373X	C	14	31	32	-	Sweden
A19/M	c.1409-1G>A	intron 10 splice site	C	28	31	32	-	Sweden
A46-1/F	c.1582_1583insGC	p.A529fs	C	13	27	27	papillary cancer, thyroid/19	Sweden
A46-2/F	c.1582_1583insGC	p.A529fs	C	18	38	40	papillary cancer, thyroid/28	Sweden
A29/M	c.1690C>T	p.R564X	C	42	-	-	-	Sweden
A34/F	c.1958G>T	exon 15 splice site	P	44	44	-	colon cancer/45	Gambia
A26/F	c.3921_3925delAAAAC	p.I1307fs	C	22	40	47	-	Sweden
A37/M	del exon 1–15	c.1-?_8532+?del	C	22	24	26	-	Sweden
A40/M	del exon 1–15	c.1-?_8532+?del	C	42	48	-	adenomyoma, gall bladder/44	Macedonia
A47/M	c.4348C>T	p.R1450X	C	20	-	-	desmoid tumor, retroperitoneum/20	Iceland
A30/F	c.5030_5031insAA	p.G1784fs	A	72	73	73	osteoma/NA three synchronous colon cancers/72	Sweden
							desmoid tumor, abdominal wall/73	
							osteoma/73 adenocarcinoma, small intestine/74	

A: attenuated FAP, P: profuse FAP, C: classical FAP

NA: not available

Attenuated FAP was demonstrated in a woman without family history of colorectal polyps or cancer. She developed synchronous colon cancers at age 72. One year later, at age 73, she was diagnosed with duodenal and gastric polyps and a desmoid tumor of the abdominal wall. The synchronous colon cancers did not lead to clinical suspicion of FAP, but the development of extracolonic features linked to FAP prompted *APC *mutation analysis, which identified an insertion (c.5030_5031insAA) in the 3'end of the gene.

## Discussion

Of the 10 previously unpublished mutations here reported, 9 were located in the part of the *APC *gene associated with classical FAP (table 1) [5,15]. New mutations are estimated to cause 20–25% of FAP and 5 cases here identified, including two individuals with whole-gene *APC *deletions, most likely represent new mutations [14,15]. The expression of colonic polyposis, i.e. profuse, classical and attenuated forms with variable ages at onset, has in several studies been linked to the genotype, whereas genotype-phenotype correlations for the extracolonic features remain to define. Upper gastrointestinal tumors include duodenal adenomas, which develop in 50–90% of individuals, fundic gland polyps in 40–50%, and adenomas in the gastric antrum found in 5–20%. In our series, all but one of the patients developed extracolonic features and an increased risk has been described for patients with mutations in exon 4, in codons 564–1465, and in the 3'end of *APC *[6,7,10]. Desmoid tumors develop in 10–15% of FAP patients and have been associated with mutations distal to codon 1444, a family history of desmoids and previous surgical trauma [16]. The two cases with desmoids here identified support this link with mutations located in codons 1450 and 1784. Several other factors, e.g. prior abdominal surgery and modifying genes, may however influence the risk. Identification of such risk-predisposing factors would be clinically valuable in order to determine the risk of desmoids tumor development after prophylactic surgery.

Extraintestinal malignancies, i.e. thyroid cancer, hepatoblastoma, and brain tumors, develop only in a few percent of FAP patients. Female FAP patients are at about 100 times increased risk of thyroid cancer, which typically develop at age 30 [17,18]. Two sisters in our series developed papillary thyroid cancer at ages 19 and 28, respectively. Although rare, thyroid cancer in first-degree relatives has been reported in a small number of families [19-21]. A link between thyroid cancer and mutations in the 5'part of exon 15 (codons 1286 and 1513) has been suggested [18]. Unfortunately, mutation data lack in most of the other familial cases reported, but two families with affected first-degree relatives and mutations 5' to codon 1513 have been reported. Our finding of thyroid cancer in two sisters with the insertion c.1582_1583insGC pushes the thyroid cancer associated region of *APC *further 3', but until the genetic factors that cause thyroid cancer in FAP have been delineated, caution is warranted for at-risk females in families where thyroid cancer has been diagnosed.

AFAP with development of less than 100 colonic polyps and a higher age at onset is estimated to represent 10% of the cases [22]. The first symptom, i.e. development of colon cancer at age 72 in an individual with a mutation in codon 1784, i.e. within the 3' part of exon 15, reflects the high risk of colon cancer despite a low number of polyps in AFAP [9,23,24]. This woman also developed a desmoid tumor and an osteoma at age 73 and an adenocarcinoma of the small intestine at age 74. Mutations beyond codon 1600 are rare, but may be underestimated with currently used mutation detection techniques. This case thus serves as a reminder of the need to consider FAP in patients who develop synchronous colorectal cancers and extracolonic features also at older age.

## Competing interests

The authors declare that they have no competing interests.

## Authors' contributions

MN evaluated the genetic analyses, collected clinical data and wrote the article. UK performed genetic counseling and was responsible for family data. ME, OJ and PM were responsible for clinical data and identified relevant patients for genetic testing. ER performed the APC sequencing analyses. All authors read and approved the final manuscript.

## Pre-publication history

The pre-publication history for this paper can be accessed here:


